# Prospective changes in diastolic function in patients with rheumatoid arthritis

**DOI:** 10.1186/s13075-022-02864-0

**Published:** 2022-08-05

**Authors:** Elizabeth Park, Kazato Ito, Rabia Iqbal, Isabelle Amigues, Sabahat Bokhari, Jennifer Van Eyk, Christopher Depender, Jon T. Giles, Joan Bathon

**Affiliations:** 1 Division of Rheumatology, Department of Medicine, Columbia University Irving Medical Center/New York Presbyterian Hospital, 630 W 168th St, P&S 3-450, New York, NY 10032 USA; 2 Division of Cardiology, Columbia University Vagelos College of Physicians and Surgeons and New York Presbyterian Hospital, New York, NY USA; 3 Division of Rheumatology, National Jewish Health, Denver, CO USA; 4 Lehigh Valley Heart and Vascular Institute, Allentown, PA USA; 5 Department of Biomedical Sciences, Cedars-Sinai Medical Center, Los Angeles, CA USA

**Keywords:** Rheumatoid arthritis, Heart failure, Diastolic dysfunction, Disease activity

## Abstract

**Background:**

Diastolic dysfunction (DD) is more prevalent in patients with rheumatoid arthritis (RA) compared to the general population. However, its evolution over time and its significant clinical predictors remain uncharacterized. We report on baseline and prospective changes in diastolic function and its associated RA and cardiovascular (CV) predictors.

**Methods:**

In this study, 158 RA patients without clinical CV disease (CVD) were enrolled and followed up at 4 to 6 years, undergoing baseline and follow-up echocardiography to assess for DD, as well as extensive characterization of RA disease activity and CV risk factors. Novel measures of myocardial inflammation and perfusion were obtained at baseline only. Using baseline and follow-up composite DD (E/e′, Left Atrial Volume Index (LAVI) or peak tricuspid regurgitation (TR) velocity; ≥ 1 in top 25%) as the outcome, multivariable regression models were constructed to identify predictors of DD.

**Results:**

DD was prevalent in RA patients without clinical heart failure (HF) (40.7% at baseline) and significantly progressed on follow-up (to 57.9%). Baseline composite DD was associated with baseline RA disease activity (Clinical Disease Activity Index; CDAI) (OR 1.39; 95% CI 1.02–1.90; *p*=0.034). Several individual diastolic parameters (baseline E/e′ and LAVI) were associated with troponin-I and brain natriuretic peptide (BNP). Baseline and follow-up composite DD, however, were not associated with myocardial inflammation, myocardial microvascular dysfunction, or subclinical atherosclerosis.

**Conclusions:**

DD is prevalent in RA patients without clinical HF and increases to >50% over time. Higher RA disease activity at baseline predicted baseline composite DD. Future longitudinal studies should explore whether adverse changes in diastolic function lead to clinical HF and are attenuated by disease-modifying antirheumatic drugs (DMARDs).

**Supplementary Information:**

The online version contains supplementary material available at 10.1186/s13075-022-02864-0.

## Background

Individuals with rheumatoid arthritis (RA) are at a 1.5 to 2-fold higher risk of heart failure (HF) vs non-RA controls. While HF phenotyping studies are scarce in RA, HF with preserved ejection fraction (HFpEF) (formerly diastolic HF) appears to be as common as HF with reduced ejection fraction (HFrEF) in RA [1]. Some of the higher HF risk stems from a higher rate of ischemic events in RA due to accelerated coronary artery disease (CAD) [2, 3]. However, even when adjusting for CAD, the two-fold risk of HF in RA persists, indicating that not all RA associated HF is ischemic in nature. Therefore, non-ischemic inflammatory pathways are likely a major contributor to HFpEF risk in RA patients [4–6].

Left ventricular (LV) diastolic dysfunction (DD), characterized by impaired relaxation and elevated LV chamber filling pressures, is thought to be a precursor to HFpEF. This trajectory is supported by two observations in the general population: Clinical HF is preceded by an asymptomatic period of diastolic decline [7] and more severe DD constitutes a higher risk for clinical HF [8]. The prevalence of DD has been investigated in RA patients without clinical HF. A meta-analysis of cross-sectional echocardiographic studies in RA patients vs non-RA controls, both without clinical HF, revealed a higher prevalence of DD in RA. However, prospective studies investigating the progression from DD to clinical HF are lacking.

RA is hypothesized to promote DD through pro-inflammatory pathways that result in subclinical myocardial fibrosis and stiffness [6, 9]. In several cross-sectional studies, an association between RA duration and key diastolic parameters was reported [10–13]. Only two prospective studies [14, 15] of diastolic function in RA patients (without clinical HF) have been published. In these, correlations between prospective changes in diastolic function and self-reported disease activity as well as with several markers of inflammation (C-reactive protein; CRP; interleukin-6; IL-6) were reported. However, objective measures of joint inflammation were not assessed, and no studies have examined the association of local (myocardial) inflammation and/or myocardial microvascular dysfunction with the presence or progression of DD in RA.

In this study, we raise the following: (1) are objective measures of RA disease activity associated with baseline DD and/or its progression?; (2) are myocardial inflammation and/or microvascular dysfunction associated with baseline DD and its progression?; and (3) do validated CV biomarkers of myocardial injury and dysfunction identify RA patients with baseline DD and predict its progression? We utilized the RHeumatoid Arthritis studY of THe Myocardium (RHYTHM) cohort [16, 17] to explore these questions, since these RA patients, who were without clinical CVD, underwent a comprehensive cardiac assessment at baseline, including echocardiography (for assessment of LV structure and function), cardiac positron emission tomography–computed tomography (PET-CT) (for assessment of myocardial inflammation, myocardial blood flow and coronary artery calcium), and measurement of inflammatory and cardiac biomarkers. Moreover, a subset of the cohort returned approximately five years later for repeat echocardiography, allowing a re-assessment of LV structure and function. Here we report the analysis of baseline and longitudinal follow-up of diastolic function, as well as its significant predictors.

## Patients and methods

### Patients

The RHYTHM cohort [16, 17] was originally designed as a cross-sectional study in 2011 to identify myocardial phenotypes in RA patients without clinical CVD and then extended to include a follow-up visit 4–6 years later to study echocardiographic changes in LV structure and function. Inclusion criteria were age ≥ 18 years and fulfillment of the American College of Rheumatology/European League Against Rheumatism 2010 classification criteria for RA [18]. Exclusion criteria included any prior self-reported physician-diagnosed CV event or procedure, contraindication to pharmacologic stress agents, and active cancer. The cohort consisted of 158 patients who completed the baseline visit and 60 who returned for follow-up. The baseline visit consisted of clinical questionnaires, examination of the joints for tenderness and swelling, biospecimen collection, echocardiography, and cardiac PET-CT scan. Echocardiography was repeated at follow-up. The study was approved by the Columbia University Irving Medical Center, New York Presbyterian Hospital Institutional Review Board. All subjects provided written informed consent prior to enrollment.

### Clinical characteristics

#### Demographic and cardiovascular characteristics

The assessment of demographics, lifestyle characteristics, blood pressure, and RA and non-RA medications were performed as previously described [16, 17]. RA disease duration was assessed by patient self-report of the date of diagnosis. Forty-four joints were examined for swelling and tenderness by the same trained assessor. RA disease activity was calculated with the Disease Activity Score for 28 joints using C-reactive protein (DAS28-CRP) and with the Clinical Disease Activity Index (CDAI). The Health Assessment Questionnaire (HAQ) was used as a measure of self-reported disability [19].

### Imaging

#### Echocardiography

Transthoracic 2-dimensional (2D) echocardiography was performed using a commercially available system (iE 33; Philips Ultrasound, Bothell, WA) by a registered cardiac sonographer according to a standardized protocol. Using apical 2 and 4-chamber views, as well as real-time 3-dimensional (3D) echocardiography, LAVI measures were obtained.

Using pulsed-wave Doppler, the peak E wave velocity, deceleration time (DT) of E wave of mitral inflow, and the peak e′ velocities of the septal and lateral mitral annulus were measured. The E/e′ ratio was calculated as an index of LV filling pressure. The peak tricuspid regurgitation (TR) velocity was measured by continuous-wave Doppler in apical 4 chamber view. For consistency for the purposes of these analyses, all baseline (*n* = 158) and follow-up (*n*= 60) 3D diastolic parameters were re-read by the same echocardiographer (KI) without blinding to time sequence. Table 1 summarizes each diastolic parameter and its echocardiographic definition as well as physiological implications.

**Table 1:** Diastolic parameters TTE definition and physiologic implications

***Diastolic parameter***	***TTE definition***	***Physiologic implications***
**Mitral E wave velocity**	Peak modal velocity across the mitral valve in early diastole	If high, indicates impaired LV relaxation + elevated LV filling pressures
**DT of E wave**	Deceleration time of mitral E velocity from peak to the baseline	If low/shortened, impaired LV relaxation or elevated LV filling pressures
**Septal e’ wave velocity** **Lateral e′ wave velocity**	Tissue Doppler-based measure of LV muscle relaxation at septal/lateral aspect of mitral valve annulus, in early diastole	If low, indicates impaired LV relaxation
**E/e′**	Mitral E wave velocity divided by e′ (averaged from septal/lateral e′)	Corrected for the effect of LV relaxation; If high, indicates elevated LV filling pressures
**LA volume index**	Left atrial volume indexed to BSA	If high, indicates cumulative effects of elevated LV filling pressures
**TR velocity**	Peak tricuspid valve regurgitant velocity	Estimation of pulmonary artery systolic pressure; If high, indicates elevated LV filling pressures

#### Cardiac FDG PET-CT

Cardiac PET-CT scans were performed at baseline in order to measure myocardial inflammation using ^18^F-Fluorodeoxyglucose (FDG), rest-stress myocardial perfusion using 13N-ammonia, and coronary artery calcium (CAC).

1. The protocol for measuring and quantifying myocardial FDG uptake (standardized uptake value; SUV), as well as the reconstruction of cardiac axes and segments, have been described extensively in a prior publication [17]. Patients followed a high fat/no carbohydrate and caffeine/methylxanthine free diet for 24 h prior to the scan and fasted for 12 h prior to the scan.
2. The detailed protocol for measuring myocardial blood flow pre- and post-vasodilation with adenosine or regadenoson has also been previously described in detail [16]. Reduced myocardial flow reserve (MFR; ratio of the myocardial blood flow (MBF)) measured at maximal vasodilation over the MBF at rest; defined as < 2.5) reflects myocardial microvascular dysfunction, in the absence of flow limiting coronary artery disease [20].
3. CAC was assessed as a measure of macrovascular (coronary artery) atherosclerosis and quantified using the Agatston method [21]. The presence of CAC was defined as an Agatston score of greater than zero.

### Laboratory measurements

Phlebotomy was performed on the morning of the PET-CT scan after an overnight fast. Sera and plasma were separated by centrifugation and frozen at −80°C. Details of laboratory measurements have been previously published [16, 17]. Seropositivity for rheumatoid factor and anti-CCP (anticyclic citrullinated peptide antibody) was defined at ≥ 40 units and ≥ 60 units, respectively. The levels of cholesterol (HDL, LDL, triglycerides), C-reactive protein (CRP), glucose, insulin, IL (interleukin)-6, BNP, and galectin-3 were measured in the Biomarkers Core Laboratory of the Columbia University Clinical and Translational Research Center.

Troponin-I levels were undetectable in baseline RHYTHM sera by the Architect STAT assay (Abbot Laboratories) and subsequently re-measured via the high-sensitive Quanterix Simoa^TM^ Human Troponin-I 2.0 immunoassay.

### Statistical analysis

The cut-offs for normal values for each individual diastolic parameter (septal/lateral e’ wave, E/e′, TR velocity, LAVI; baseline and follow-up) were referenced from the American Society of Echocardiography (ASE)/ European Association of Cardiovascular Imaging (EACVI) echocardiographic criteria [22]. Each individual diastolic parameter (listed above and additionally E wave and DT of E wave for historical significance) was also examined as a continuous outcome. ASE/EACVI criteria define diastolic dysfunction (DD) as having 2 or more out of 4 abnormal diastolic parameters (E/e′, septal/lateral e′ velocity, TR velocity, and/or LAVI). ASE/EACVI criteria focus on symptomatic DD while the RHYTHM cohort was selected for absence of known cardiac disease. Therefore, to assess putative earlier stages of DD, we also assessed the prevalence of one or more abnormal diastolic parameters (baseline and follow-up). Finally, we explored a composite definition of DD as having absolute values of one or more of the following criteria—E/e′, LAVI, or peak TR velocity—in the top quartile of the group as a whole. We constructed regression models based on this composite definition to explore predictors of composite DD at baseline and follow-up.

Means and standard deviations (SDs) for normally distributed variables, and counts and percentages for categorical variables, were calculated. For continuous variables, differences were compared using Student’s *t*-tests or the Wilcoxon-Mann-Whitney test/signed ranks. Categorical variables were compared using the *χ*^2^ goodness of fit test, Fisher’s exact test, or McNemar’s test, as appropriate.

Linear regression was used to model the associations of clinical and laboratory characteristics with primary outcomes (composite DD; individual diastolic parameters). Multivariable models were constructed by including any variable associated with the outcome (*p*<0.25) in univariable models, and a *p*-value of <0.05 was considered significant for final models. All multivariable models were examined for co-linearity, omitted variables, and outliers. All analyses were performed using Stata version 16 (StataCorp, College Station, TX).

## Results

### Patient characteristics

The characteristics of the full cohort at baseline (*n*=158) and the subset that returned for follow-up (*n*=60) are summarized in Table 2. The baseline cohort was middle aged, predominantly female and multi-ethnic (41% Hispanic). Mean RA disease activity was in the moderate range. Twenty-nine percent were on TNF inhibitors, 65% on methotrexate, and 30% on prednisone. Cardiovascular risk factors were also prevalent at baseline, with 39% of patients reporting a history of smoking, 34% using blood pressure medications, and 15% using a statin. Additionally, 17% of patients had a CAC score ≥ 100, while the mean CAC of those with a score > 0 was 130. Lastly, reduced MFR (< 2.5) was demonstrated in 25% of patients and abnormal (high) myocardial FDG uptake (cutoff > 3.10 units as previously defined) [17] in 17% as well. In the subset that returned for follow-up, shorter RA duration, higher frequency of methotrexate use, and higher mean CRP level were noted. Statistically significant changes from baseline to follow-up in the subset included decreases in DAS28CRP and IL-6 levels, less frequent use of methotrexate, and an increase in mean systolic blood pressure (SBP).

**Table 2:** Baseline characteristics of the RHYTHM full cohort and subset

Characteristic	Full cohort baseline (***n***=158)	Longitudinal cohort at baseline (***n***=60)	Longitudinal cohort at follow-up (***n***=60)
**Demographic**			
**Age**	54±12	53 ±11	57±12
**Female (158)**	133 (84)	49 (82)	49 (82)
**Race/ethnicity (150)**			
**White**	54 (36)	24 (40)	
**Black**	27 (18)	10 (17)	
**Hispanic**	62 (41.3)	24 (40)	
**Other**	7 (4.67)	2 (3.3)	
**RA characteristics**			
**Disease duration (years)**	10.7 ± 11.9	8.3 ± 9.6	ND
**CDAI**	17.5 ± 12.4	16.7 ± 12.3	13.6 ± 12
**DAS28-CRP**	3.71 ± 1.36	3.26 ± 1.1	3.1 ± 1.3**
**RF or anti-CCP (% positive) (145)**	107 (73.3)	41 (69.5)	ND
**CRP per mg/liter**	5.1 ± 7.5	6.1 ± 9.4	7.1 ± 18.8*
**IL-6 (log) per mg/liter**	1.1 ± 1.2	1.2 ± 1.1	0.84 ± 1.1*
**BNP per pg/mL**	21.8 ± 17.8	21.7 ± 20.6	ND
**Troponin-I (pg/mL)**	1.08 ± 1.56	0.90 ± 1.3	ND
**Galectin-3 (ng/mL)**	9.56 ± 4.86	9.64 ± 5.6	ND
**RA medication**			
**No DMARDs**	15 (9.6)	3 (5)	8 (13)
**MTX (156)**	102 (65)	46 (77)	25 (42)**
**Targeted DMARDs (156)**	64 (41)	25 (42)	35 (58)
**TNF inhibitor**	45 (29)	19 (30)	21 (35)
**Prednisone (156)**	47 (30.1)	15 (25)	4 (7.3)
**CV risk factors**			
**Current smoker (155)**	16 (10.3)	6 (10)	4 (7.1)
**Ever smoker (155)**	60 (38.7)	22 (36.7)	ND
**SBP mm/Hg (149)**	118 ±16.7	116 ±16.8	124 ±17.0**
**BP medications (156)**	53 (34)	12 (20)	13 (23)
**Statin (156)**	23 (14.7)	12 (20)	13 (22.8)
**Total cholesterol (145)**	194 ± 37.9	187 ± 33	186.3 ±30.8
**LDL (145)**	112 ± 33.1	107.2 ± 29.9	103.6 ± 27.5
**Diabetes**^**#**^**(149)**	13 (7.9)	4 (7.4)	8 (14.3)
**PET/CT cardiac measures**			
**CAC score (157)**			
**0**	106 (67.9)	44 (75)	ND
**≥ 100**	27 (17.2)	10 (17)	ND
**Mean myocardial SUV, mean (149)**	2.54±2.04	2.7±2.2	ND
**Max myocardial SUV, mean (148)**	3.95±3.29	4.5±3.6	ND
**Myocardial flow reserve (111)**	2.92±0.69	2.87± 0.62	ND

*p<0.05 **p<0.01; baseline subset (*n*=60) vs follow up subset (*n*=60)

*ND* not done

(*n*) Under “Characteristic” column indicates total number of patients with variable measurements

#Diabetes was defined as a fasting serum glucose level of ≥ 126 mg/dl or use of antidiabetic medications

Continuous values are expressed as mean± SD and categorical values are expressed as *n* (%)

### Diastolic function

Although RHYTHM patients at baseline were without clinical signs or symptoms of HF, we assessed whether any met ASE/ EACVI echocardiographic criteria [22] for symptomatic DD (septal and lateral e’ velocity, E/e′ ratio, peak TR velocity, LAVI) at baseline or at follow-up (Table 3).

**Table 3:** Prevalence of diastolic dysfunction in the RHYTHM full cohort and longitudinal subset

Diastolic parameters	Full cohort baseline (***n***=158)	Longitudinal subset at baseline (***n***=60)	Longitudinal subset at follow-up (***n***=60)	Baseline vs follow-up in longitudinal subset, ***p***-value*
**By ASE/EACVI criteria**				
0 abnormal parameter^**^n(%)	77 (49)	35 (59.3)	28 (43.7)	***0.02***
**≥ 1 abnormal parameter*n*(%)**	78 (49.7)	24 (40.7)	33 (57.9)	***0.02***
**Individual diastolic parameters (%)**				
**E/e′ > 14**	3.2	0	3.4	…
**Septal e’ wave velocity < 7**	26	10	32	***0.07***
**Lateral e’ wave velocity < 10**	37	21	46	***0.045***
**TR velocity > 2.8**	6.5	2.8	2.8	...
**LA volume index > 34**	6.9	3.3	5.6	0.56

*McNemar’s test

**Abnormal E/e′, septal/lateral e’ wave velocity, TR velocity, or LA volume index (developed for general population with symptoms of dyspnea or other HF symptoms); Supplementary Table 4 with additional definitions

All values expressed are %, unless otherwise indicated

Using these established cut-offs, the frequency of patients fulfilling > 1 criterion at baseline was 40.7% and increased significantly to 57.9% at follow-up. Additionally, 21% of patients fulfilled > 2 criterion at baseline and 26% at follow-up. The percentages of patients who met each individual criterion at the two visits are also shown in Table 3. The prevalence of abnormal peak septal e’ velocity (< 7 cm/s) and the peak lateral e’ velocity (< 10cm/s) increased (*p*=0.07 and *p*=0.045, respectively) from baseline to follow-up. There were non-significant trends of increasing prevalence of abnormal E/e′ ratio and LAVI as well.

Absolute values at baseline and follow-up for each individual diastolic parameter and the annualized rates of change are summarized in Supplementary Table 1 and Supplementary Table 2, respectively. A statistically significant decline in lateral e’ velocity and increase in TR velocity were observed, indicating worsening diastolic function. The mean raw percentage changes in each parameter from baseline to follow-up ranged from 1 to 9.3% (Supplementary Table 2).

Though not an outcome of this study, mean ejection fraction (EF) was normal at baseline (62.7% ± 4.67 (mean ±SD)) and remained preserved on follow-up (60.4% ± 4.32).

### Univariable and multivariable analyses

Univariable and multivariable models were used to identify variables associated with abnormal diastolic function at baseline and follow-up. For these analyses, a composite outcome measure defined as having one or more diastolic variables (E/e′ ratio, LAVI, or peak TR velocity) in the top quartile (Table 4) was constructed. We also constructed MV analyses for several of the individual DD criteria (Supplementary Tables 5, 6 and 7).

**Table 4:** Univariable and multivariable associations of RA participant characteristics with baseline composite diastolic dysfunction*

	Univariable (***n***=158)			Multivariable (***n***=131)		
Demographics (baseline)	OR	95% CI	***p***-value	OR	95% CI	***p***-value
**Age, per year**	***1.09***	***1.05–1.12***	***<0.01***	***1.08***	***1.03–1.14***	***0.003***
**Male versus female**	1.36	0.58–3.21	0.48	0.45	0.12–1.70	0.24
**Race/ethnicity**						
**White**	REF	REF				
**Black**	0.59	0.23–1.51	0.27			
**Hispanic**	1	0.48–2.07	1.00	----	----	----
**Other**	0.40	0.071–2.24	0.30			
**BMI, per kg/m**^**2**^	1.03	0.98–1.08	0.26	0.96	0.89–1.04	0.36
**RA characteristics (baseline)**						
**RA duration (square root), per year**	***1.25***	***1.02–1.52***	***0.031***	1.19	0.90–1.57	0.23
**Joint deformities (square root)**	**1.72**	**1.31–2.26**	***<0.01***	----	----	----
**CDAI (square root), per unit**	***1.24***	***1.01–1.53***	***0.037***	***1.39***	***1.02–1.90***	***0.034***
**DAS28CRP**	**1.29**	**1.01–1.65**	***0.0041***	----	----	----
**RF (baseline)**						
**0: <15 (REF)**	REF	REF		REF	REF	REF
**1: 15–500**	2.94	0.77–11.25	0.12	3.50	0.59–20.70	0.17
**2: >500**	4.44	0.94–21.00	0.060	4.44	0.58–33.88	0.15
**CCP (baseline)**						
**0: <15.6 (REF)**	REF	REF	REF			
**1: 15.6–250**	2.11	0.50–8.95	0.31	----	----	----
**2: >250**	2.1	0.50–8.73	0.31			
**Square root CRP, per mg/liter**	1.24	0.95–1.61	0.11	----	----	----
**Log IL-6, per mg/liter**	1.19	0.90–1.58	0.21	0.88	0.58–1.33	0.54
**Log BNP, per pg/mL**	1.54	0.77–3.08	0.22	----	----	----
**Log troponin-I, per pg/mL**	***1.84***	***1.32–2.56***	***<0.01***	1.097	0.66–1.81	0.72
**Log galectin-3 ng/mL**	1.91	0.89–4.10	**0.097**	----	----	----
**RA medication**						
**NSAIDs**	1.14	0.60–2.18	0.68	----	----	----
**Prednisone**	1.08	0.54–2.14	0.83	----	----	----
**Leflunomide**	***4.48***	***1.18–16.98***	***0.027***	***7.18***	***1.19–43.4***	***0.032***
**Methotrexate**	1.07	0.55–2.07	0.84	----	----	----
**TNF inhibitor**	0.94	0.47–1.89	0.86	----	----	----
**Tocilizumab**	0.59	0.053–6.68	0.67	----	----	----
**CV risk factors (baseline)**						
**Current smoker, yes versus no**	1.24	0.44–3.50	0.68	----	----	----
**Ever smoker, yes versus no**	1.53	0.80–2.94	0.20	1.17	0.48–2.84	0.73
**SBP (baseline), mm/Hg**	***1.03***	***1.01–1.05***	***0.003***	1.00	0.98–1.03	0.71
**Statin use, yes versus no**	1.37	0.56–3.32	0.49	----	----	----
**Total cholesterol, per mg/dL**	1.00	0.99–1.01	0.62	----	----	----
**LDL, per mg/dL**	1.00	0.99–1.01	0.88	----	----	----
**Square root HDL, per mg/dL**	1.08	0.82–1.43	0.57	----	----	----
**PET/CT cardiac measures (baseline)**						
**CAC score>100**	***4.42***	***1.75–11.22***	***0.002***	----	----	----
**CAC score>300**	***5.63***	***1.52–20.8***	***0.010***	6.76	0.73–62.23	0.091
**Log mean myocardial SUV, per unit**	1.52	0.83–2.77	0.17	----	----	----
**Log max myocardial SUV, per unit**	**1.64**	**0.97–2.71**	**0.066**	1.37	0.72–2.60	0.34
**Myocardial flow reserve (MFR)**	0.75	0.43–1.30	0.31	----	----	----
**Prob>F**				***<0.001***		
**Pseudo R-Squared**				0.26		

*Baseline diastolic dysfunction defined as having ≥ 1 of E/e′, LAVI, or TR Vmax in top 25%

#### Association of demographic and RA characteristics with diastolic function

**Fig. 1:**
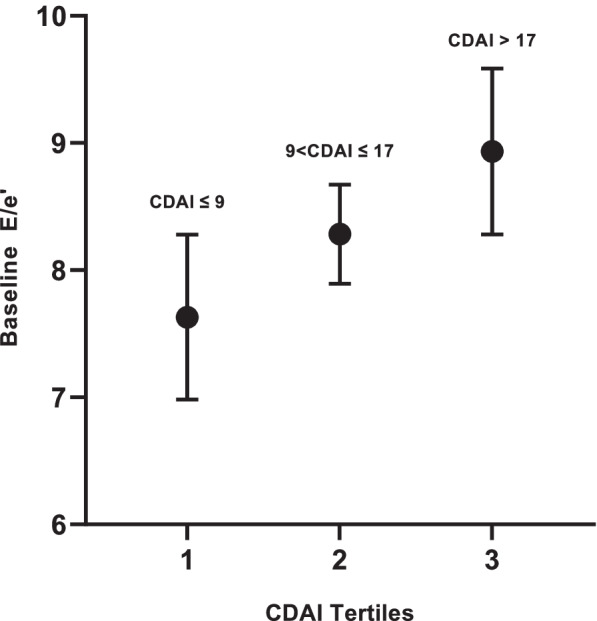
Adjusted associations of CDAI tertiles with baseline E/e′. Adjusted means and 95% CIs are depicted. All adjusted analyses account for age, gender, BMI, and troponin-I

In univariable analyses, higher age and leflunomide use were associated with baseline diastolic function (Table 4, Supplementary Table 3, 5 and 7). In a multivariable model adjusted for relevant covariates, CDAI was significantly associated with baseline composite DD (OR 1.39; 95% CI 1.02–1.90; *p=0.*034) (Table 4), as was DAS28CRP in a separate multivariable model (OR 1.46; 95% CI 1.01–2.11; *p=0.046*) (Supplementary Table 3). Additionally, the use of leflunomide was associated with baseline composite DD (OR 7.18; 95% CI 1.19–43.4; *p=0.032*) (Table 4). However, neither baseline CDAI nor averaged CDAI (baseline + follow-up) was a significant predictor of follow-up composite DD (OR 1.02; 95% CI 0.94–1.12; *p*=0.57) (Supplementary Table 4). In multivariable models using individual diastolic parameters as outcomes, baseline CDAI was associated with baseline E/e′ ratio (Fig. 1; Supplementary Table 5), and with baseline LAVI (Supplementary Table 7).

#### Associations of myocardial inflammation, MFR, and CV risk factors, with diastolic function

In univariable analyses, myocardial SUV, CAC scores, and SBP were consistently associated with baseline diastolic function (Table 4, Supplementary Tables 3, 4, 5 and 7). However, in multivariable models, MFR, myocardial SUV, SBP, and CAC score > 300 were no longer associated with either baseline composite DD (Table 4) or with follow-up composite DD (Supplementary Table 4). There were no consistent associations of smoking status or cholesterol levels with the composite DD outcome, nor with most of the individual DD outcomes (Table 4; Supplementary Table 3, 4, 5, 6 and 7). However, significant associations between SBP and baseline and annualized rate of change in E/e′ were noted in multivariable analyses (Supplementary Table 5 and 6, respectively). Additionally, MFR was significantly associated with one diastolic parameter (baseline LAVI) (Supplementary Table 7).

#### Associations of biomarkers of myocardial injury with diastolic function

**Fig. 2:**
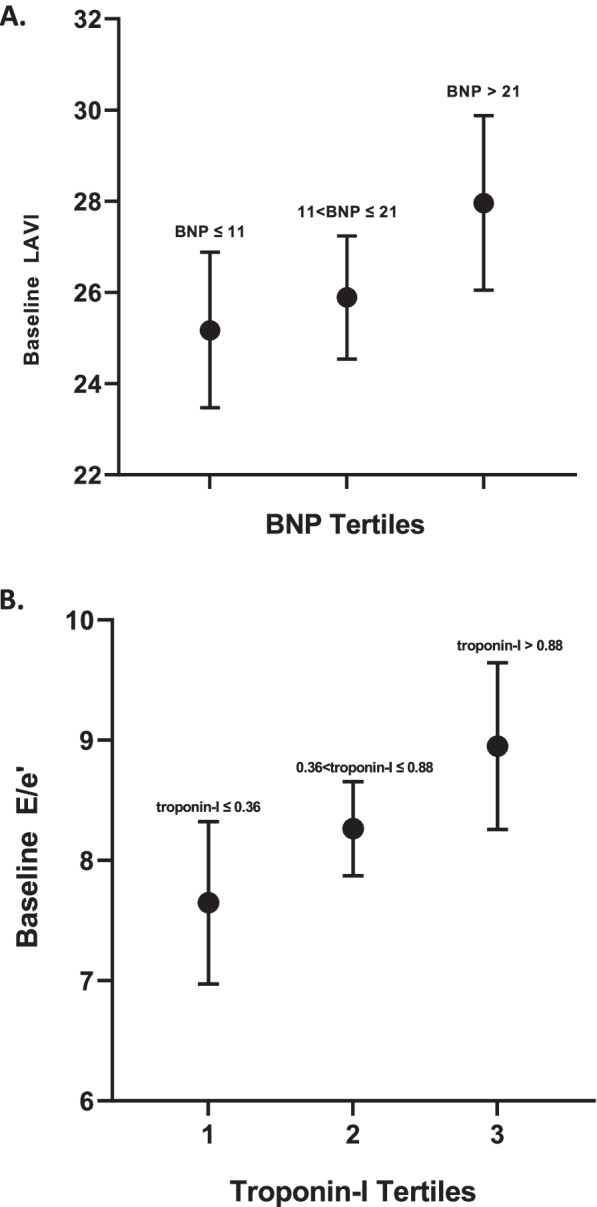
Adjusted associations of **A** BNP tertiles with baseline LAVI. **B** Troponin-I Tertiles with baseline E/e′. Adjusted means and 95% CIs are depicted; all adjusted analyses account for age, gender, and BMI

In univariable analyses, troponin and galectin-3 were significantly associated with baseline E/e′ (Supplementary Table 5), while BNP and troponin were significantly associated with baseline LAVI (Supplementary Table 7). In multivariable models, BNP was not a significant predictor of baseline composite DD (Supplementary Table 3). However, BNP (> 21) was associated with baseline LAVI, independent of RA and CV covariates (Fig. 2A) (Supplementary Table 7). In multivariable models, troponin-I was not a significant predictor of baseline composite DD (Table 4). However, troponin-I (> 0.88) was associated with baseline E/e′ ratio, while adjusting for RA and CV covariates, including CDAI (Fig. 2B) (Supplementary Table 5).

## Discussion

The current study enriches and extends previous cross-sectional studies of DD in RA patients, by examination of a cohort with comprehensive multi-modal CV phenotyping and with longitudinal follow-up in a subset. The major findings of our study are that DD was prevalent in RA patients without clinical HF, progresses on follow-up, and is associated with RA disease activity and with several cardiac biomarkers, but not with myocardial inflammation, myocardial microvascular dysfunction, or with subclinical atherosclerosis.

Our finding of a baseline frequency of DD of 40.7%, defined by at least one criterion for DD (and 21% by ≥ 2 criterion), is consistent with previous reports citing prevalences between 26 and 49% [16, 23, 24]. In our study, this frequency increased to 57.9% over an interval of 3–5 years. Direct comparisons of frequencies may be misleading due to utilization of different criteria. We utilized the updated, arguably more stringent, 2016 ASE/EACVI criteria for DD which exclude DT, E/A, and isovolumic relaxation time (IVRT) that were utilized in many of the prior studies and were considered to have poor reproducibility, including our previous publication [16].

In our longitudinal analyses, septal/lateral e′ wave velocities (average to form e′) declined significantly, while the prevalence of abnormal septal e′ (< 7) and lateral e′ (< 10) increased significantly, all together indicating worsening diastolic function. We also observed non-statistically significant increases in E/e′ and LAVI, also consistent with worsening DD. To our knowledge, there are only two prior longitudinal studies of DD in RA [14, 15]. Our observed increase in E/e′ and trend towards increase in LAVI are consistent with that of Davis et al. [14]. Other contributory trends towards worsening DD included a significant increase in TR velocity, which was not analyzed in the other two studies.

Risk factors for the development of DD in the general population include age and hypertension, as well as mildly inflammatory states such as obesity and insulin resistance [7, 8, 25, 26]. Concordantly in our study, we observed a consistently positive association between age and multiple individual baseline diastolic parameters (E/e′, and LAVI,) as well as with baseline composite DD. Higher SBP was associated with higher baseline E/e′ and annualized rate of change in E/e′, but not with composite DD, in our patient cohort. These results reinforce the importance of tight management of conventional CV risk factors for the prevention of CVD in RA patients. Also, elevated calcium scores (CAC > 100), though prevalent (17% at baseline), were not significantly associated with composite baseline or follow-up composite DD, underscoring the point that DD can occur independently of atherosclerosis.

RA-associated inflammation is more severe than that of other chronic inflammatory conditions that are associated with DD and HFpEF, such as obesity and diabetes, and is hypothesized to be the primary driver of the higher prevalence of DD observed in RA vs the general population [27]. To examine this hypothesis, we assessed the correlation of multiple measures of inflammation in our RA cohort with DD. Whereas prior studies of DD in RA utilized only serum markers of inflammation and/or patient self-reported measures of joint pain [13, 14], ours is the first study to include physical assessments of swollen and tender joint counts in our assessment of disease activity and to use cardiac PET-CT to assess local (myocardial) inflammation and perfusion, in addition to serum markers of inflammation, to assess the relationship of RA disease activity with DD. While individual markers like ESR, CRP, and IL-6 did not associate with baseline or follow-up composite DD (only CRP was associated with annualized rate of change in E/e′; Supplementary Table 6), measures of RA disease activity that do (DAS28CRP) or do not (CDAI) incorporate CRP were both positively associated with baseline composite DD, as well as with multiple individual baseline parameters of DD (E/e′ and LAVI), even after adjusting for smoking, blood pressure, cholesterol levels, and CAC levels, suggesting that inflammation contributes to DD independently of conventional CV risk factors. However, neither averaged CDAI nor DAS28CRP was a predictor of follow-up DD, possibly owing to the relatively small number of patients who returned for follow-up, and the possibility that this effect may take longer than 5 years to observe. The previous literature demonstrated correlations of diastolic function with RA duration [10–12] which we did not observe. Another, albeit indirect, way to probe the impact of disease activity on DD is to explore whether specific RA DMARD treatment(s), through subduing of disease activity, are associated with a reduced risk of DD. Interestingly, we did note a strong positive association between leflunomide use and baseline composite DD (Table 4), but given the limited number of patients who were using leflunomide at baseline (13/156) and statistical uncertainty (wide confidence interval), meaningful interpretation is limited and this observation should be confirmed in additional studies.

Our study is one of few in the literature that analyzed sensitive biomarkers of myocardial injury, including BNP and troponin-I, with respect to diastolic function. BNP and troponin-I were associated with individual parameters of diastolic function (LAVI and E/e′, respectively), but not baseline composite DD. BNP is released in response to atrial contraction and increased LA pressure; thus, its positive association with LAVI (Supplementary Table 7), a surrogate measure of LA pressure, appears to be physiologically consistent. Troponin has long been heralded as a marker of myocyte injury/necrosis and prognosticator of CV associated death in non-RA patients [28]. In our patients, higher troponin-I levels were associated with higher E/e′ (and thus higher LV filling pressures), indicating a possible link between subclinical myocardial damage and diastolic filling. These results suggest that cardiac biomarkers may be used in conjunction with echocardiography to identify patients at risk for DD and HFpEF, however this will require formal evaluation in future studies.

Subclinical myocarditis is prevalent in RA, as evidenced by myocardial inflammation in up to 20–40% of RA patients without clinical CVD as assessed by cardiac FDG PET/CT [17] or by late gadolinium uptake on cardiac magnetic resonance (CMR) imaging in other RA cohorts [29–32]. It is postulated that persistent myocardial inflammation leads to LV wall fibrosis and stiffness (with concurrent myocardial endothelial damage, microvascular dysfunction and reduced perfusion) and then altered LV function [33]. Surprisingly, we found no association in multivariable analyses of myocardial mean or max SUV with baseline or follow-up composite DD. Since most studies of myocardial inflammation report imaging findings at only one time point, it is unclear whether myocardial inflammation is transient or persistent and recurrent. If transient, it may not constitute a significant contributor to the development of DD. We previously reported that 29% of the RHYTHM RA cohort had reduced MFR even after adjusting for CAC scores and that lower MFR was associated with higher LAVI but not E/e′ at baseline [16]. In the present studies, we did not identify MFR as a predictor of worsening DD over time.

The main strengths of this study are the large baseline sample size (> 150) of RA patients without clinical CVD, and the extensive cardiac phenotyping that included a comprehensive set of echocardiography measures, novel measures of myocardial inflammation and /perfusion, cardiac biomarkers, a measure of atherosclerosis, and follow-up echocardiography in a significant number of the original cohort. Limitations include the absence of matched non-RA controls that preclude direct comparisons, but previous data confirm a higher rate of DD in RA vs non-RA without clinical HF. While we incorporated change in RA disease activity over time by averaging disease activity scores in regression models, as well as any change in RA DMARD treatment on follow-up, treatment was not assigned or randomized, which limits our interpretation of these findings as associations but not causality. Although the number of patients who returned for follow-up was limited (about a 1/3 of the baseline cohort), we did note significant declines in several key diastolic parameters.

## Conclusions

In conclusion, DD is prevalent in RA patients without clinical HF and increases to > 50% over time. Adverse changes in diastolic function are associated with higher RA disease activity, suggesting that tight control of RA disease activity could attenuate the onset or progression of DD and possibly of HFpEF. Given the twofold higher mortality rate associated with HF in RA vs non-RA [3], early detection and monitoring of DD in RA, determining whether DD clearly predicts development of clinical HF, and whether DD is reversible or attenuated by DMARDs, should be explored further in longitudinal, controlled studies.

## Supplementary Information

**Additional file 1. **Supplementary Data. Description of data: % and annualized rate of change in diastolic parameters, univariable/multivariable associations of composite follow-up DD and individual diastolic parameters. **Table 1**. Change in measures of diastolic function in the RHYTHM follow-up subset. **Table 2**. Percentage and annualized rate of change in the RHYTHM follow-up subset. **Table 3**. Univariable and multivariable associations of RA participant characteristics with baseline composite diastolic dysfunction. **Table 4**. Univariable and multivariable associations of RA participant characteristics with follow-up composite diastolic dysfunction. **Table 5**. Univariable and multivariable associations of RA, CV factors with baseline E/e’. **Table 6**. Univariable and multivariable associations of RA, CV factors with annualized rate of ▲ in E/e’. **Table 7**. Univariable and multivariable associations of RA, CV factors with baseline LAVI.

## Data Availability

The datasets used and analyzed during the current study are included in this published article (and its supplementary information files) or otherwise available from the corresponding author on request.

## Abbreviations

2D: 2-Dimensional
3D: 3-Dimensional
Anti-CCP: Anticyclic citrullinated peptide antibody
ASE/EACVI: American Society of Echocardiography/European Association of Cardiovascular Imaging Echocardiographic Criteria
BNP: Brain natriuretic peptide
CAC: Coronary artery calcium
CAD: Coronary artery disease
CDAI: Clinical Disease Activity Index
CMR: Cardiac magnetic resonance
CRP: C-reactive protein
CV: Cardiovascular
CVD: Cardiovascular disease
DAS28-CRP: Disease Activity Score for 28 joints using CRP
DD: Diastolic dysfunction
DMARDs: Disease-modifying antirheumatic drugs
DT: Deceleration time
FDG: 18F-Fluorodeoxyglucose
HAQ: Health Assessment Questionnaire
HF: Heart failure
HFpEF: HF with preserved ejection fraction
HFrEF: HF with reduced ejection fraction
IL-6: Interleukin-6
LAVI: Left Atrial Volume Index
LV: Left ventricular
MBF: Myocardial blood flow
MFR: Myocardial flow reserve
PET-CT: Positron emission tomography–computed tomography
RA: Rheumatoid arthritis
RHYTHM: RHeumatoid Arthritis studY of THe Myocardium
SUV: Standardized uptake value
TR: Tricuspid regurgitation
