# Supplementary material for: Prospective changes in diastolic function in patients with rheumatoid arthritis
Source: Arthritis Res Ther. 2022 Aug 5;24:184. doi: 10.1186/s13075-022-02864-0 (PMC9354314; doi:10.1186/s13075-022-02864-0)
Supplement: Supplementary file 1 — Additional file 1. Supplementary Data. Description of data: % and annualized rate of change in diastolic parameters, univariable/multivariable associations of composite follow-up DD and individual diastolic parameters. Table 1. Change in measures of diastolic function in the RHYTHM follow-up subset. Table 2. Percentage and annualized rate of change in the RHYTHM follow-up subset. Table 3. Univariable and multivariable associations of RA participant characteristics with baseline composite diastolic dysfunction. Table 4. Univariable and multivariable associations of RA participant characteristics with follow-up composite diastolic dysfunction. Table 5. Univariable and multivariable associations of RA, CV factors with baseline E/e’. Table 6. Univariable and multivariable associations of RA, CV factors with annualized rate of ▲ in E/e’. Table 7. Univariable and multivariable associations of RA, CV factors with baseline LAVI. [file 13075_2022_2864_MOESM1_ESM.docx]

**Supplementary Data**

**Table 1. Change in measures of diastolic function in** **the RHYTHM follow-up subset**

| **Diastolic Parameters**  **(mean ±SD)** | **Full Cohort Baseline (n=158) %** | **Longitudinal subset at baseline (n=60) %** | **Longitudinal subset at follow-up (n=60) %** | **p-value** |
| --- | --- | --- | --- | --- |
| **Septal e’ wave velocity** | 8.03±2.19 | 8.22± 2.19 | 7.65±1.72 | <0.10 |
| **Lateral e’ wave velocity** | 10.8±2.78 | 11.3± 2.60 | 10.4±2.17 | ***<0.05*** |
| **E wave velocity** | 75.1± 15.8 | 71.4± 15.7 | 66.8±17.2 | <0.10 |
| **E/e’^#^** | 8.33±2.47 | 7.55± 1.94 | 7.89±2.75 | ns |
| **TR velocity** | 2.3± 0.38 | 1.87± 1.01 | 2.2± 0.48 | ***<0.05*** |
| **LA volume index** | 24.8 ± 5.53 | 22.5± 5.19 | 24.3± 5.23 | ns |
| **DT of E wave** | 230±53.3 | 230± 55.28 | 245±65.8 | ns |

**#e’ represents an averaged value of the septal averaged septal/lateral e’ wave velocities**

**p-value denoting significance of change between longitudinal subset at baseline and longitudinal subset at follow-up parameters; ns= not significant**

**Table 2. Percentage and annualized rate of change in the RHYTHM follow-up subset**

| **Diastolic Parameters**  **(mean ±SD)** | **Mean % change between baseline and follow-up** | **Mean annualized rate of change of follow-up (SD)** |
| --- | --- | --- |
| **E/e’^#^** | +7.73 | **+0.094** **(0.68)** |
| **Septal e’ wave velocity** | -3.79 | **-0.12** **(0.46)^*^** |
| **Lateral e’ wave velocity** | -6.20 | **-0.23** **(0.46)^**^** |
| **DT of E wave** | +9.3 | **+2.56** **(20.2)** |
| **TR velocity** | -5.4 | **-0.038(0.10)^**^** |
| **LA volume index** | -1.16 | **-0.15 (1.22)** |
| **E wave velocity** | -3.97 | **-1.05 (3.91)^*^** |

**# represents an averaged value of the septal averaged septal/lateral e’ wave velocities**

****p<0.10 **p<0.05***

**Table 3. Univariable and multivariable associations of RA participant characteristics with baseline composite diastolic dysfunction***

|  | **Univariable (n=158)** | | | **Multivariable (n=125)** | | | **Multivariable (n=92)** | | |
| --- | --- | --- | --- | --- | --- | --- | --- | --- | --- |
| **Demographics(baseline)** | **OR** | **95% CI** | **p-value** | **OR** | **95% CI** | **p-value** | **OR** | **95% CI** | **p-value** |
| **Age, per year** | ***1.09*** | ***1.05-1.12*** | ***<0.01*** | **1.09** | **1.03-1.15** | ***0.004*** | ***1.08*** | ***1.02-1.14*** | ***0.005*** |
| **Male versus female** | 1.36 | 0.58-3.21 | 0.48 | 0.77 | 0.20-2.96 | 0.71 | 0.59 | 0.14-2.52 | 0.48 |
| **Race/ethnicity**  **White**  **Black**  **Hispanic**  **Other** | REF  0.59  1.0  0.40 | REF  0.23-1.51  0.48-2.07  0.071-2.24 | REF  0.27  1.00  0.30 | REF  0.49  0.68  ---- | REF  0.13-1.83  0.22-2.05  ---- | REF  0.29  0.49  ---- | REF  0.30  0.58  ---- | REF  0.055-1.67  0.16-2.08  ---- | REF  0.17  0.40  ---- |
| **BMI, per kg/m^2^** | 1.03 | 0.98-1.08 | 0.26 | 0.95 | 0.88-1.03 | 0.26 | 0.95 | 0.86-1.05 | 0.34 |
| **RA Characteristics (baseline)** |  |  |  |  |  |  |  |  |  |
| **RA Duration (square root), per year** | ***1.25*** | ***1.02-1.52*** | ***0.031*** | **1.29** | **0.96-1.72** | **0.087** | 1.22 | 0.86-1.74 | 0.27 |
| **Joint deformities (square root)** | **1.72** | **1.31-2.26** | ***<0.01*** | ---- | ---- | ---- | ---- | ---- | ---- |
| **CDAI (square root), per unit** | ***1.24*** | ***1.01-1.53*** | ***0.037*** | ---- | ---- | ---- | ***1.47*** | ***1.01-2.15*** | ***0.044*** |
| **DAS28CRP** | **1.29** | **1.01-1.65** | **0.041** | **1.46** | **1.01-2.11** | ***0.046*** | ---- | ---- | ---- |
| **RF (baseline)**  **0: <15 (REF)**  **1: 15-500**  **2: >500** | REF  2.94  4.44 | REF  0.77-11.25  0.94-21.00 | 0.12  0.060 | REF  3.43  **8.39** | REF  0.59-19.84  **0.99-71.3** | REF  0.20  ***0.051*** | REF  1.11  2.83 | REF  0.0017-727.16  0.0038-2112.87 | REF  0.97  0.76 |
| **CCP (baseline)**  **0: <15.6 (REF)**  **1: 15.6-250**  **2: >250** | REF  2.11  2.1 | REF  0.50-8.95  0.50-8.73 | REF  0.31  0.31 | ---- | ---- | ---- | ---- | ---- | ---- |
| **Square root CRP, per mg/liter** | **1.26** | **0.97-1.63** | **0.078** | ---- | ---- | ---- | ---- | ---- | ---- |
| **Log IL-6, per mg/liter** | 1.19 | 0.90-1.58 | 0.21 | ---- | ---- | ---- | ---- | ---- | ---- |
| **Log BNP, per pg/mL** | 1.54 | 0.77-3.08 | 0.22 | ---- | ---- | ---- | 1.21 | 0.49-3.02 | 0.68 |
| **Log troponin-I, per pg/mL** | ***1.84*** | ***1.32-2.56*** | ***<0.01*** | 1.09 | 0.65-1.85 | 0.74 | ---- | ---- | ---- |
| **Log galectin-3 ng/mL** | 1.91 | 0.89-4.10 | **0.097** | ---- | ---- | ---- | ---- | ---- | ---- |
| **RA Medication** |  |  |  |  |  |  |  |  |  |
| **NSAIDs** | 1.14 | 0.60-2.18 | 0.68 | ---- | ---- | ---- | ---- | ---- | ---- |
| **Prednisone** | 1.08 | 0.54-2.14 | 0.83 | ---- | ---- | ---- | ---- | ---- | ---- |
| **Leflunomide** | ***4.48*** | ***1.18-16.98*** | ***0.027*** | ***4.89*** | ***0.76-31.3*** | ***0.094*** | 2.54 | 0.32-19.85 | 0.37 |
| **Methotrexate** | 1.07 | 0.55-2.07 | 0.84 | ---- | ---- | ---- | ---- | ---- | ---- |
| **TNF inhibitor** | 0.94 | 0.47-1.89 | 0.86 | ---- | ---- | ---- | ---- | ---- | ---- |
| **Tocilizumab** | 0.59 | 0.053-6.68 | 0.67 | ---- | ---- | ---- | ---- | ---- | ---- |
| **CV Risk Factors (baseline)** |  |  |  |  |  |  |  |  |  |
| **Current smoker, yes versus no** | 1.24 | 0.44-3.50 | 0.68 | ---- | ---- | ---- | ---- | ---- | ---- |
| **Ever smoker, yes versus no** | 1.53 | 0.80-2.94 | 0.20 | 1.00 | 0.39-2.61 | 0.99 | 1.38 | 0.42-4.56 | 0.59 |
| **SBP (baseline), mm/Hg** | ***1.03*** | ***1.01-1.05*** | ***0.003*** | 1.01 | 0.98-1.04 | 0.54 | 1.00 | 0.97-1.04 | 0.89 |
| **Statin use, yes versus no** | 1.37 | 0.56-3.32 | 0.49 | ---- | ---- | ---- | ---- | ---- | ---- |
| **Total cholesterol, per mg/dL** | 1.00 | 0.99-1.01 | 0.62 | ---- | ---- | ---- | ---- | ---- | ---- |
| **LDL, per mg/dL** | 1.00 | 0.99-1.01 | 0.88 | ---- | ---- | ---- | ---- | ---- | ---- |
| **Square root HDL, per mg/dL** | 1.08 | 0.82-1.43 | 0.57 | ---- | ---- | ---- | ---- | ---- | ---- |
| **PET/CT Cardiac Measures (baseline)** |  |  |  |  |  |  |  |  |  |
| **CAC score>100**  **CAC score>300** | ***4.42***  ***5.63*** | ***1.75-11.22***  ***1.52-20.8*** | ***0.002***  ***0.010*** | 1.54  ---- | 0.33-7.07  ---- | 0.58  ---- | 3.07  ---- | 0.40-23.3  ---- | 0.28  ---- |
| **Log mean Myocardial SUV, per unit** | 1.52 | 0.83-2.77 | 0.17 | ---- | ---- | ---- | ---- | ---- | ---- |
| **Log max myocardial SUV, per unit** | 1.64 | 0.97-2.71 | **0.066** | 1.50 | 0.78-2.88 | 0.23 | 1.40 | 0.59-3.32 | 0.44 |
| **MFR** | 0.75 | 0.43-1.30 | 0.31 | ---- | ---- | ---- | ---- | ---- | ---- |
| **Prob>F**  **Pseudo R-Squared** |  | | | ***0.0001***  **0.26** | | | ***0.0066***  **0.25** | | |

***Baseline diastolic dysfunction defined as having ≥ 1 of E/e’, LAVI, or TR Vmax in top 25%**

**Table 4. Univariable and multivariable associations of RA participant characteristics with follow-up composite diastolic dysfunction***

|  | **Univariable (n=57)** | | | **Multivariable (n=51)** | | | **Multivariable (n=44)** | | |
| --- | --- | --- | --- | --- | --- | --- | --- | --- | --- |
| **Demographics (baseline)** | **OR** | **95% CI** | **p-value** | **OR** | **95% CI** | **p-value** | **OR** | **95% CI** | **p-value** |
| **Age, per year** | 1.01 | 0.96-1.05 | 0.72 | 0.98 | 0.93-1.05 | 0.69 | 0.98 | 0.91-1.05 | 0.61 |
| **Male versus female** | 0.82 | 0.20-3.31 | 0.79 | 0.82 | 0.11-5.99 | 0.84 | 0.60 | 0.058-6.15 | 0.67 |
| **Race**  **White**  **Black**  **Hispanic**  **Other** | REF  1.75  0.97  1.4 | REF  0.37-8.20  0.30-3.14  0.078-25.14 | REF  0.48  0.96  0.82 | REF  Non-white  1.17 | REF  Non-white  0.22-6.15 | 0.85 | REF  Non-white  1.098 | REF  Non-white  0.19-6.24 | REF  0.92 |
| **BMI, per kg/m^2^** | 0.97 | 0.88-1.07 | 0.56 | 0.96 | 0.84-1.08 | 0.49 | 0.95 | 0.82-1.10 | 0.50 |
| **RA characteristics (baseline)** |  |  |  |  |  |  |  |  |  |
| **RA Duration (square root), per year** | 1.32 | 0.90-1.96 | 0.16 | ---- | ---- | ---- | ---- | ---- | ---- |
| **Joint deformities (square root)** | 1.35 | 0.89-2.06 | 0.15 | ---- | ---- | ---- | ---- | ---- | ---- |
| **Baseline DAS28CRP** | 1.03 | 0.69-1.55 | 0.86 | ---- | ---- | ---- | ---- | ---- | ---- |
| **AveragedDAS28CRP (baseline + fu)** | 0.90 | 0.55-1.46 | 0.67 | ---- | ---- | ---- | ---- | ---- | ---- |
| **Baseline CDAI (square root)** | 1.07 | 0.78-1.47 | 0.67 | ---- | ---- | ---- | ---- | ---- | ---- |
| **Averaged CDAI**  **(baseline + fu)** | 1.01 | 0.96-1.06 | 0.79 | 1.02 | 0.94-1.12 | 0.57 | 1.04 | 0.93-1.15 | 0.49 |
| **RF (baseline)**  **0: <15 (REF)**  **1: 15-500**  **2: >500** | REF  …  1.87 | REF  …  0.37-9.27 | REF  …  0.44 | ---- | ---- | ---- | ---- | ---- | ---- |
| **CCP (baseline)**  **>250** | 1.43 | 0.49-4.18 | 0.51 | ---- | ---- | ---- | ---- | ---- | ---- |
| **Square root CRP, per mg/liter** | 1.25 | 0.88-1.76 | 0.21 | 1.34 | 0.81-2.21 | 0.26 | 1.58 | 0.81-3.09 | 0.18 |
| **Log IL-6, per mg/liter** | 1.21 | 0.75-1.95 | 0.43 | ---- | ---- | ---- | ---- | ---- | ---- |
| **Log BNP, per pg/mL** | 0.59 | 0.22-1.56 | 0.29 | ---- | ---- | ---- | ---- | ---- | ---- |
| **Log troponin-I, per pg/mL** | 1.18 | 0.72-1.96 | 0.51 | ---- | ---- | ---- | ---- | ---- | ---- |
| **Log galectin-3 ng/mL** | 0.89 | 0.29-2.74 | 0.83 | ---- | ---- | ---- | ---- | ---- | ---- |
| **RA Medication** |  |  |  |  |  |  |  |  |  |
| **NSAID use, y vs no** | 0.39 | 0.12-1.34 | 0.14 | 0.28 | 0.0599-1.28 | 0.10 | **0.21** | **0.036-1.20** | **0.079** |
| **Prednisone use, y vs no** | 1.33 | 0.17-10.25 | 0.78 | ---- | ---- | ---- | ---- | ---- | ---- |
| **Methotrexate use, y vs no** | 0.50 | 0.17-1.50 | 0.22 | ---- | ---- | ---- | ---- | ---- | ---- |
| **TNF inhibitor use, y vs no** | 1.02 | 0.34-3.07 | 0.97 | ---- | ---- | ---- | ---- | ---- | ---- |
| **Tocilizumab use, y vs no** | 0.82 | 0.12-5.34 | 0.83 | ---- | ---- | ---- | ---- | ---- | ---- |
| **CV Risk Factors (baseline)** |  |  |  |  |  |  |  |  |  |
| **Current smoker, y vs no** | 2.67 | 0.23-31.41 | 0.44 | ---- | ---- | ---- | ---- | ---- | ---- |
| **Ever smoker, y vs no** | 1.07 | 0.36-3.21 | 0.90 | ---- | ---- | ---- | ---- | ---- | ---- |
| **SBP (baseline), mm/Hg** | 0.99 | 0.96-1.03 | 0.78 | ---- | ---- | ---- | ---- | ---- | ---- |
| **SBP (follow-up), mm/Hg** | 1.01 | 0.97-1.04 | 0.70 | ---- | ---- | ---- | ---- | ---- | ---- |
| **DBP (baseline), mm/Hg** | 1.00 | 0.93-1.07 | 0.99 | ---- | ---- | ---- | ---- | ---- | ---- |
| **Statin use, yes versus no** | 0.91 | 0.25-3.36 | 0.89 | ---- | ---- | ---- | ---- | ---- | ---- |
| **Total cholesterol, per mg/dL** | 0.999 | 0.98-1.02 | 0.91 | ---- | ---- | ---- | ---- | ---- | ---- |
| **LDL, per mg/dL** | 0.98 | 0.98-1.02 | 0.79 | ---- | ---- | ---- | ---- | ---- | ---- |
| **Square root HDL, per mg/dL** | 1.00 | 0.97-1.03 | 0.83 | ---- | ---- | ---- | ---- | ---- | ---- |
| **PET/CT Cardiac Measures (baseline)** |  |  |  |  |  |  |  |  |  |
| **CAC score>100**  **CAC score>300** | 1.35  4.23 | 0.34-5.30  0.41-43.37 | 0.67  0.22 | ----  8.78 | ----  0.33-232.88 | ----  0.19 | ----  21.63 | ----  0.30-1550.36 | ----  0.16 |
| **Log mean Myocardial SUV, per unit** | 0.47 | 0.16-1.39 | 0.18 | 0.38 | 0.087-1.65 | 0.19 | 0.46 | 0.11-1.83 | 0.27 |
| **Log max myocardial SUV, per unit** | 0.56 | 0.23-1.36 | 0.199 | ---- | ---- | ---- | ---- | ---- | ---- |
| **MFR** | 0.97 | 0.34-2.77 | 0.95 | ---- | ---- | ---- | ---- | ---- | ---- |
| **Prob>F** |  | | | 0.38 | | | 0.35 | | |
| **Pseudo R-Squared** |  | | | 0.14 | | | 0.18 | | |

***Follow-up diastolic dysfunction defined as having** **≥ 1 of E/e’, LAVI, or TRvmax in top 25%**

**Table 5. Univariable and multivariable associations of RA, CV factors with baseline E/e’**

|  | **Univariable (n=155)** | | **Multivariable (n=127)** | |
| --- | --- | --- | --- | --- |
| **Demographics (baseline)** | **β** | **P** | **β** | **P** |
| **Age, per year** | **0.063** | ***<0.01*** | **0.040** | **0.080** |
| **Male versus female** | -0.54 | 0.32 | **-1.36** | ***0.028*** |
| **Race**  **White**  **Black**  **Hispanic**  **Other** | REF  0.78  0.45  -0.67 | REF  0.19  0.33  0.50 | 0.47  -0.16  0.18 | 0.44  0.76  0.88 |
| **BMI, per kg/m^2^** | **0.091** | ***0.006*** | -0.0087 | 0.81 |
| **RA Characteristics (baseline)** |  |  |  |  |
| **RA duration (square root), per year** | **0.26** | ***0.029*** | ---- | ---- |
| **Joint deformities (square root)** | **0.30** | ***0.057*** | ---- | ---- |
| **CDAI (square root), per unit** | **0.40** | ***0.001*** | ***0.37*** | ***0.012*** |
| **DAS28CRP, per unit** | **0.51** | ***0.001*** | ---- | ---- |
| **RF**  **0: <15 (REF)**  **1: 15-500**  **2: >500** | REF  -0.22  0.83 | REF  0.76  0.34 | ---- | ---- |
| **CCP**  **0: <15.6 (REF)**  **1: 15.6-250**  **2: >250** | REF  **-1.47**  -1.22 | REF  **0.082**  0.14 | REF  **-1.58**  **-1.48** | REF  **0.059**  **0.075** |
| **Square root CRP, per mg/L** | **0.42** | ***0.010*** | 0.097 | 0.58 |
| **Log IL-6, per pg/mL** | **0.31** | **0.073** | ---- | ---- |
| **Log BNP, per pg/mL** | 0.27 | 0.53 | ---- | ---- |
| **Log troponin-I, per pg/mL** | **0.75** | ***<0.01*** | **0.46** | ***0.040*** |
| **Log galectin-3, per ng/mL** | **0.94** | ***0.043*** | ---- | ---- |
| **RA Medication** |  |  |  |  |
| **NSAID use, yes versus no** | **0.78** | **0.058** | **0.98** | ***0.030*** |
| **Prednisone use, yes versus no** | 0.66 | 0.13 | ---- | ---- |
| **Methotrexate use, yes versus no** | -0.55 | 0.20 | ---- | ---- |
| **Tocilizumab use, yes versus no** | 1.44 | 0.32 | ---- | ---- |
| **TNF inhibitor use, yes versus no** | 0.14 | 0.75 | ---- | ---- |
| **CV Risk Factors (baseline)** |  |  |  |  |
| **Current smoker, yes versus no** | **1.17** | **0.082** | 0.089 | 0.90 |
| **Ever smoker, yes versus no** | **0.11** | ***0.013*** | ---- | ---- |
| **SBP (baseline), mm/Hg** | **0.051** | ***<0.01*** | **0.036** | ***0.007*** |
| **DBP (baseline), mm/Hg** | **0.043** | ***0.044*** | ---- | ---- |
| **Statin use, yes versus no** | 0.74 | 0.19 | ---- | ---- |
| **Total cholesterol, per mg/dl** | -0.0024 | 0.66 | ---- | ---- |
| **LDL, per mg/dl** | -0.0024 | 0.70 | ---- | ---- |
| **Square root HDL, per mg/dl** | -0.25 | 0.15 | ---- | ---- |
| **PET/CT Cardiac Measures (baseline)** |  |  |  |  |
| **CAC score>100**  **CAC score>300** | 0.54  **1.33** | 0.31  ***0.048*** | ----  0.46 | ----  0.56 |
| **Log mean Myocardial SUV, per unit** | 0.52 | 0.18 | 0.21 | 0.58 |
| **Log max myocardial SUV, per unit** | 0.39 | 0.25 | ---- | ---- |
| **MFR** | -0.050 | 0.89 | ---- | ---- |
| **Prob>F** |  |  | ***<0.01*** | |
| **R-Squared** |  |  | **0.36** | |
| **Adjusted R-Squared** |  |  | **0.27** | |

**Table 6. Univariable and multivariable associations of RA, CV factors with annualized rate of ▲ in E/e’**

|  | **Univariable (n=56)** | | **Multivariable (n=54)** | |
| --- | --- | --- | --- | --- |
| **Demographics (baseline)** | **β** | **P** | **β** | **P** |
| **Age, per year** | 0.00094 | 0.91 | 0.011 | 0.29 |
| **Male versus female** | 0.0099 | 0.97 | 0.26 | 0.32 |
| **Race**  **White**  **Black**  **Hispanic**  **Other** | REF  -0.31  0.30  0.35 | REF  0.22  0.13  0.60 | REF  -0.42  0.24  0.11 | REF  0.16  0.32  0.87 |
| **BMI, per kg/m^2^** | 0.0035 | 0.83 | 0.0033 | 0.86 |
| **RA Factors** |  |  |  |  |
| **RA Duration (square root), per year** | -0.032 | 0.61 | ---- | ---- |
| **Joint deformities (square root)** | 0.073 | 0.28 | ---- | ---- |
| **DAS28CRP (follow-up)** | -0.017 | 0.77 | ---- | ---- |
| **Averaged DAS28CRP (baseline + fu)** | 0.0076 | 0.91 | ---- | ---- |
| **CDAI (square root) (follow-up)** | 0.0073 | 0.88 | ---- | ---- |
| **Averaged CDAI (baseline + fu)** | 0.0034 | 0.65 | ---- | ---- |
| **RF (baseline)**  **0: <15 (REF)**  **1: 15-500**  **2: >500** | REF  …  -0.23 | REF  …  0.41 | ---- | ---- |
| **CCP (baseline)**  **0: <15.6 (REF)**  **1: 15.6-250**  **2: >250** | REF  0.35  0.46 | REF  0.63  0.52 | ---- | ---- |
| **Square root CRP, per mg/liter** | **0.088** | ***0.05*** | **0.16** | ***0.015*** |
| **Log IL-6, per mg/liter** | 0.10 | 0.23 | ---- | ---- |
| **Log BNP (baseline), per pg/mL** | -0.19 | 0.16 | ---- | ---- |
| **Log troponin-I (baseline), per pg/mL** | -0.076 | 0.39 | -0.14 | 0.21 |
| **Log galectin-3 (baseline), per ng/mL** | 0.25 | 0.22 | ---- | ---- |
| **RA Medication** |  |  |  |  |
| **NSAID use, yes versus no** | 0.040 | 0.84 | ---- | ---- |
| **Prednisone use, yes versus no** | -0.0079 | 0.98 | ---- | ---- |
| **Leflunomide use, yes versus no** | 0.47 | 0.11 | ---- | ---- |
| **Methotrexate use, yes versus no** | -0.09 | 0.62 | ---- | ---- |
| **TNF inhibitors use, yes versus no** | -0.011 | 0.96 | ---- | ---- |
| **Tocilizumab use, yes versus no** | -0.32 | 0.25 | ---- | ---- |
| **CV Risk Factors** |  |  |  |  |
| **Current smoker, yes versus no** | -0.49 | *0.10* | -0.27 | 0.48 |
| **SBP (baseline), per mm/Hg** | -0.0075 | 0.16 | **-0.010** | **0.081** |
| **SBP (follow-up), per mm/Hg** | -0.0081 | 0.16 | ---- | ---- |
| **DBP (baseline), per mm/Hg** | -0.015 | 0.19 | ---- | ---- |
| **Statin use, yes versus no** | 0.047 | 0.80 | ---- | ---- |
| **ASA use, yes versus no** | -0.074 | 0.69 | ---- | ---- |
| **Total cholesterol, per mg/dl** | -0.0020 | 0.50 | ---- | ---- |
| **LDL, per mg/dl** | -0.0046 | 0.16 | ---- | ---- |
| **HDL, per mg/dl** | 0.0061 | *0.24* | 0.00029 | 0.96 |
| **Log triglycerides, per mg/dl** | 0.015 | 0.95 | ---- | ---- |
| **Glucose, per mg/dl** | -0.0021 | 0.68 | ---- | ---- |
| **Square root insulin, per uIU/mL** | -0.00038 | 0.86 | ---- | ---- |
|  |  |  |  |  |
| **PET/CT Cardiac Measures (baseline)** |  |  |  |  |
| **CAC score>100**  **CAC score>300** | 0.091  -0.097 | 0.72  0.79 | ---- | ---- |
| **Log max myocardial SUV, per unit** | -0.11 | 0.36 | ---- | ---- |
| **MFR** | 0.14 | 0.32 | ---- | ---- |
| **Prob>F** |  | | ***0.099*** | |
| **R-Squared** |  | | **0.31** | |
| **Adjusted R-Squared** |  | | **0.13** | |

**Table 7. Univariable and multivariable associations of RA, CV factors with baseline LAVI**

|  | **Univariable (n=146)** | | **Multivariable (n=72)** | | **Multivariable (n=125)** | |
| --- | --- | --- | --- | --- | --- | --- |
| **Demographics (baseline)** | **β** | **P** | **β** | **P** | **β** | **P** |
| **Age, per year** | **0.096** | ***0.013*** | **0.14** | ***0.020*** | ***0.15*** | ***<0.001*** |
| **Male versus female** | 0.77 | 0.54 | 2.39 | 0.24 | 1.93 | 0.15 |
| **Race**  **White**  **Black**  **Hispanic**  **Other** | REF  -1.08  -0.18  -2.90 | REF  0.44  0.87  0.23 | REF  -0.73  1.84  -0.99 | REF  0.70  0.25  0.81 | REF  -1.43  0.42  -0.86 | REF  0.30  0.73  0.75 |
| **BMI, per kg/m^2^** | -0.044 | 0.57 | 0.20 | 0.17 | 0.003 | 0.95 |
| **RA Characteristics (baseline)** |  |  |  |  |  |  |
| **RA Duration (square root), per year** | -0.20 | 0.47 | ---- | ---- | ---- | ---- |
| **Joint deformities (square root)** | 0.52 | 0.14 | ---- | ---- | ---- | ---- |
| **CDAI (square root), per unit** | 0.46 | 0.11 | ---- | ---- | ***0.79*** | ***0.018*** |
| **DAS28CRP** | 0.30 | 0.40 | ---- | ---- | ---- | ---- |
| **RF (baseline)**  **0: <15 (REF)**  **1: 15-500**  **2: >500** | REF  1.14  2.82 | REF  0.50  0.17 | REF  5.86  8.66 | REF  0.29  0.14 | REF  2.02  ***4.55*** | REF  0.20  ***0.024*** |
| **CCP (baseline)**  **0: <15.6 (REF)**  **1: 15.6-250**  **2: >250** | REF  -0.22  2.05 | REF  0.91  0.27 | ---- | ---- | ---- | ---- |
| **Square root CRP, per mg/liter** | -0.24 | 0.52 | ---- | ---- | ---- | ---- |
| **Log IL-6, per mg/liter** | 0.12 | 0.77 | ---- | ---- | ---- | ---- |
| **Log BNP, per pg/mL** | **2.70** | ***0.007*** | **2.12** | ***0.046*** | ---- | ---- |
| **Log troponin-I, per pg/mL** | **1.07** | ***0.014*** | ---- | ---- | ---- | ---- |
| **Log galectin-3 ng/mL** | -0.24 | 0.83 | ---- | ---- | ---- | ---- |
| **RA Medication** |  |  |  |  |  |  |
| **NSAID use, yes versus no** | 0.32 | 0.73 | ---- | ---- | ---- | ---- |
| **Prednisone use, yes versus no** | 0.75 | 0.46 | ---- | ---- | ---- | ---- |
| **Leflunomide use, yes versus no** | **-4.32** | ***0.023*** | ---- | ---- | ---- | ---- |
| **Methotrexate use, yes versus no** | 0.61 | 0.53 | ---- | ---- | ---- | ---- |
| **Tocilizumab use, yes versus no** | 5.13 | 0.19 | ---- | ---- | ---- | ---- |
| **TNF inhibitors use, yes versus no** | 1.18 | 0.25 | **2.71** | **0.078** | 1.28 | 0.23 |
| **CV Risk Factors (baseline)** |  |  |  |  |  |  |
| **Current smoker, yes versus no** | -2.23 | 0.13 | ---- | ---- | ---- | ---- |
| **Ever smoker, yes versus no** | **-1.71** | ***0.07*** | -1.82 | 0.19 | ***-2.69*** | ***0.007*** |
| **SBP (baseline), mm/Hg** | -0.023 | 0.41 | ---- | ---- | ---- | ---- |
| **DBP (baseline), mm/Hg** | -0.025 | 0.62 | ---- | ---- | ---- | ---- |
| **Statin use, yes versus no** | -1.79 | 0.18 | ---- | ---- | ---- | ---- |
| **Total cholesterol, per mg/dL** | 0.0055 | 0.66 | ---- | ---- | ---- | ---- |
| **LDL, per mg/dL** | -0.011 | 0.43 | ---- | ---- | ---- | ---- |
| **Square root HDL, per mg/dL** | **1.11** | ***0.006*** | **2.67** | ***<0.001*** | ***2.10*** | ***<0.001*** |
| **PET/CT Cardiac Measures (baseline)** |  |  |  |  |  |  |
| **CAC score>100**  **CAC score>300** | 1.78  **2.80** | 0.13  **0.064** | ---- | ---- | ---- | ---- |
| **Log mean myocardial SUV, per unit** | 0.63 | 0.46 | ---- | ---- | ---- | ---- |
| **Log max myocardial SUV, per unit** | 1.05 | 0.16 | ---- | ---- | 0.39 | 0.59 |
| **MFR** | **-2.61** | ***0.002*** | **-2.23** | ***0.024*** | ---- | ---- |
| **Prob>F** |  | | ***0.0012*** | | ***0.0001*** | |
| **R-Squared** |  | | 0.42 | | 0.31 | |
| **Adjusted R-Squared** |  | | 0.28 | | 0.23 | |
